# Goal attainment, medication adherence and guideline adherence in the treatment of hypertension and dyslipidemia in Irish populations: A systematic review and meta-analysis

**DOI:** 10.1016/j.ijcrp.2025.200364

**Published:** 2025-01-04

**Authors:** Rehab Elhiny, Linda M. O'Keeffe, Elizabeth O. Bodunde, Stephen Byrne, Maria Donovan, Margaret Bermingham

**Affiliations:** a Pharmaceutical Care Research Group, School of Pharmacy, University College Cork, Cork, Ireland; b Clinical Pharmacy Department, Faculty of Pharmacy, Minia University, Minia, Egypt; c School of Public Health, University College Cork, Cork, Ireland; d MRC Integrative Epidemiology Unit at the University of Bristol, University of Bristol, Bristol, UK; e Population Health Sciences, Bristol Medical School, University of Bristol, Bristol, UK; f The Irish Centre for Maternal and Child Health Research, University College Cork, Cork, Ireland

**Keywords:** Blood pressure goal achievements, LDL-C goal achievements, Medication adherence, Guideline adherence, Cardiovascular risk factors control

## Abstract

**Background:**

The appropriate treatment high blood pressure (BP) and low-density lipoprotein cholesterol.

(LDL-C), according to clinical guidelines, reduces a patient's risk of a cardiovascular event.

**Aim:**

This systematic review aims to evaluate the attainment of BP and LDL-C goals among the Irish population in both primary and secondary prevention of cardiovascular diseases, the level of adherence to prescribing guidelines by doctors and the level of medication adherence among patients.

**Methods:**

Five databases were searched in March 2024. Quantitative articles reporting levels of goals attainment, medication adherence or guideline adherence for LDL-C and BP among Irish adults aged ≥18 years were included. The proportion of patients attaining their LDL-Cor BP goals were statistically combined using the random effect model.

**Results:**

Following screening, 23 eligible articles were identified. The achievement of LDL-C <1.8 mmol/L was 41 % (95 % CI 31,52), compared to 69 % of people (95 % CI 62,76) reported to have achieved the less stringent goal of LDL-C < 3 mmol/L. The achievement of BP < 140/90 mmHg was 56 % (95 % CI 46,65). Medication adherence levels ranged between 27 % and 92 %. Guideline adherence findings demonstrated that not all patients who should be on lipid-lowering therapy are and that choice of antihypertensive is not always in line with the guidelines.

**Conclusion:**

Approximately one-third of deaths in Ireland annually are caused by cardiovascular disease, despite being preventable. There is room for improvement in goal attainments in people at risk of CVDs and optimization of medication adherence and guideline adherence may be beneficial in this population.

## 1 Introduction

Cardiovascular diseases (CVDs) are the leading cause of death worldwide [1]. Ireland, along with the European Union as a whole, continue to have a high prevalence of CVDs, with the prevalence of some CVDs such as hypertension increasing year on year [2]. CVDs is the second most common cause of death in Ireland in 2021, while it was still the most common cause of death in the European Union [2]. In addition, deaths from some CVDs, including hypertension, are increasing in both Ireland and the European Union [2]. Poorly controlled risk factors, in particular hypertension and dyslipidemia, are among the most primary causes of coronary heart disease and stroke [3,4]. However, attaining the guideline goals for blood pressure (BP) and low-density lipoprotein-cholesterol (LDL-C) in the real world is challenging [[5], [6], [7]]. The European Society of Cardiology (ESC)/European Atherosclerosis Society (EAS) set goals for LDL-C in the treatment of dyslipidemia, based on the overall cardiovascular risk assessment [8,9]. BP goals are generally based on the European Society of Hypertension/European Society of Cardiology (ESH/ESC) recommendations; one stringent BP goal for people with high risk or very high risk people including people who have had a previous diagnosis of myocardial infarction (MI), stroke, renal dysfunction or proteinuria, and another less stringent goal for hypertensive people generally [[10], [11], [12], [13]].

According to the Euro Aspire IV survey, of 7653 participants with CVDs across 27 European countries, 42 % failed to attain the BP goals outlined in the 2013 European Society of Hypertension/European Society of Cardiology (ESH/ESC) hypertension guidelines [7]. In the same survey, only 37 % of people with CVDs on high-intensity lipid lowering therapy and 26 % of those on low to moderate intensity lipid lowering therapy achieved an LDL-C < 1.8 mmol/L [14]. This demonstrates a low level of goal attainment for BP and LDL-C goals in a European context.

Medication non-adherence is one of the factors that contribute to therapeutic failure and the subsequent clinical consequences, especially among people with CVDs [15]. Suboptimal statin adherence is reported among people with existing CVDs [15]. In a cross-sectional study conducted in Sweden, medication adherence was measured among statin users by the Morisky Medication Adherence Scale (4-item) [16]. The study reported that among 414 participants on statin therapy; 54.5 % were classified as high adherent and 45.5 % were classified as low adherent to their statin therapy [16]. Anti-hypertensive medication adherence was reported in a systematic review of 25 studies from 19 countries, including 12,628 hypertensive participants in which 45 % were non-adherent to antihypertensive medications, and of the 2606 participants with uncontrolled hypertension, 84 % were non adherent [17]. Additionally, it has been noted that guideline non-adherence and ineffective dissemination of the guidelines among health care professionals, especially general practitioners, may negatively affect the achievement of the recommended goals [11,18]. Norwegian and German studies reported results about lipid lowering therapy utilization and its association with LDL-C goal achievements where they reported lower proportions of LDL-C goal attainments among high/very high risk people and underutilization of the lipid-lowering therapy (LLT) among people respectively [19,20]. A study in the United States (US) showed a relationship between adherence to anti-hypertensive medications and BP goal attainments [21]. However, evidence linking BP and LDL-C goal attainment, medication adherence and guideline adherence is still sparse in both an international and Irish context.

Using a systematic review and meta-analysis, the aim of this study is to quantify the levels of goal attainment, patient medication adherence and prescriber guideline adherence for lipid-lowering therapies and antihypertensives among people treated for hypertension and/or dyslipidemia (primary prevention) and people with existing cardiovascular diseases (secondary prevention) in adult populations in Ireland.

## 2 Methods

This systematic review and meta-analysis was conducted according to the protocol registered in PROSPERO (CRD42023422050) [22] and in line with The Preferred Reporting Items for Systematic Reviews and Meta-Analysis (PRISMA) 2020 checklist [23].

### 2.1 Study selection

Observational studies including adult participants aged ≥18 years with diagnosed hypertension or dyslipidemia and treated for any of these conditions in Ireland only were included in the systematic review. Studies including participants treated for secondary prevention of CVDs including acute coronary syndrome, MI and stroke with antihypertensive and lipid-lowering medications were also included. Systematic reviews, literature reviews, meta-analysis and conference abstracts were excluded. Where studies reported duplicate data, the most recent and complete study was included.

### 2.2 Search methods

A detailed search was conducted in March 2024 using the advanced search function of five databases; PubMed, Scopus, CINAHL, Embase, and Web of Science. No restrictions were placed on language or date of publications. The search strategy for each database included CVDs, patient adherence, guideline adherence and goal attainments terms as presented in the Supplementary Table 1. After removing duplicate results, one author (RE) screened the titles and abstracts of the remaining articles to identify studies for full text review. Studies eligible for full text reviewing were independently reviewed by three authors (MB, LOK & RE). Relevant data were extracted in excel spread sheets by two authors (RE & EO). Any disagreements that occurred were resolved by a discussion between the reviewers. The search results were managed using the reference management software Zotero®

### 2.3 Data extraction

Data were extracted from each study in a structured Microsoft Excel® spreadsheet. Data on the year of the execution of the study, participants demographics, sample size, study design, setting, guideline that was followed, LDL-C, total cholesterol (TC), and BP goals, prescribing recommendations, the proportions of participants who achieved LDL-C, TC and BP goals, proportion of people who utilize antihypertensive medications or LLT, assessment tools for medication adherence, and proportions of people who were reported adherent to antihypertensive medications, LLT or secondary prevention medications.

### 2.4 Risk of bias assessment

Risk of bias was assessed using the National Heart, Lung, and Blood Institute Quality Assessment Tool for Observational Cohort and Cross-sectional Studies [24]. Two authors assessed the articles, and when disagreements arose, they were resolved through discussion and referral to one of the authors for adjudication if necessary. Quality was rated according to the number of questions for which the answer was yes- 0 for poor (0–4 out of 14 questions), for fair (5–10 out of 14 questions), or for good (11–14 out of 14 questions); NA: not applicable, NR: not reported [24,25]**.**Studies were included regardless of the findings of the quality assessment, but scores of the articles selected were reported.

### 2.5 Data analysis

Proportional meta-analysis was conducted using the statistical software STATA (version 17.0). All meta-analysis used the random effects (mixed) model and presented the point estimate with 95 % confidence intervals (CIs) [26,27]. Heterogeneity was tested using I^2^ test. A meta-analysis was undertaken on studies identified in the systematic review that included the proportion of participants who attained the lipid targets and BP targets to estimate the overall proportion of participants who attained LDL-C and BP goals. Subgroup analyses was performed in which studies were grouped according to whether the participants were treated for primary or secondary prevention. Participants in the primary prevention group were those at moderate risk/high risk/very high risk of CVDs without known CVDs, or participants whose target was LDL-C < 3 mmol/L. The secondary prevention group of participants were those with known CVDs and/or whose target was LDL-C <2.5 mmol/L or LDL <1.8 mmol/L. Data about medication adherence and levels of guidelines adherence were narratively summarized and grouped; however meta-analysis was not performed due to heterogeneity of methodologies for these outcome measures.

## 3 Results

**Fig. 1:**
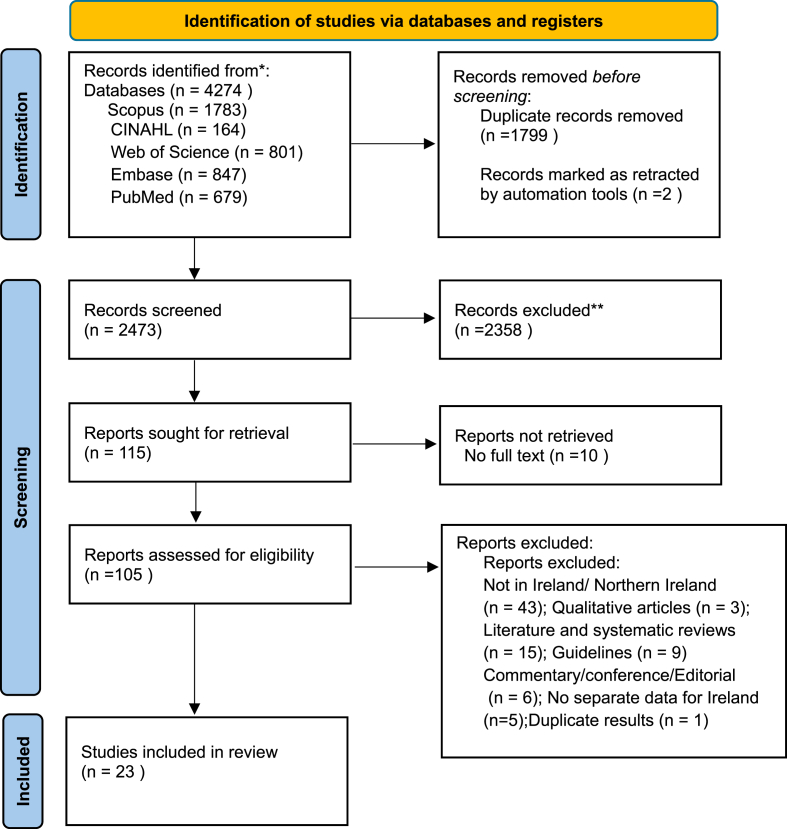
Prisma Flow diagram.

The initial databases search yielded 4274 publications. Of these, 1799 were excluded as duplicates and 2 were excluded as they had been retracted. After screening the titles and abstracts of the remaining search results, and checking the availability of the entire texts, 105 articles were selected for full text review. A total of 23 articles met the inclusion criteria (Fig. 1). All studies included in this review were cohort and cross-sectional studies. The risk of bias assessment found that 15 of the included studies were of fair quality and 8 of the included studies met the criteria for being of good quality (Supplementary Table 2).

### 3.1 Goal attainment

Twelve studies reported data about BP and lipid target attainments including LDL-C and TC on 8696 participants [5,6,11,12,[28], [29], [30], [31], [32], [33], [34], [35], [36]]. Three studies were either retrospective cohort, or retrospective chart review studies [6,29,30], two studies were prospective cohort studies [33,36], and the remaining seven studies were cross-sectional studies [5,11,12,28,31,32,35]. Six studies reported outcomes from multiple European countries including Ireland [5,12,28,[30], [31], [32]]. Three studies were conducted in community settings [11,35,36], six in hospital settings [6,12,[29], [30], [31], [32]], and three in combined hospital and community setting [5,28,33]. The proportion of people achieving clinical guideline goals was recorded in six studies for BP [6,11,12,[30], [31], [32]] and in 11 studies for cholesterol (LDL-C and/or TC) [5,6,[28], [29], [30], [31], [32], [33],[35], [36], [37]]. The mean age (± standard deviation) of participants ranged from 59 (±9.4) [12] to 71 (±13) years [33]. Participants recruited to these studies were treated for both primary prevention and secondary prevention [6,11,28,29,35,36] or secondary prevention of CVDs alone [12,[30], [31], [32], [33]]. Primary prevention including studies that recruited people with no existing cardiovascular diseases [6,11,36] and/or people who had been stratified to very high risk, high risk and low (no) risk based on their CVD risk [5,28,35]. Table 1.

**Table 1:** Study Characteristics and Goal Attainment of Lipid and BP targets (12 studies).

							Recommended guideline goals			Proportion of participants achieving guideline goals		
Study/Year	Quality assessment	Participants demographics	CVD (in secondary prevention cases)	Design	Setting	Guidelines	BP	LDL-C	TC	BP	LDL-C	TC
(EUROASPIRE II Study Group), 2001	∗∗	Age ≤70, N = 345; secondary prevention participants	Acute MI/Acute myocardial ischemia	Cross-Sectional	Hospital	Second Joint European Societies Task Force (1998)	<140/90 (mmHg)		<5 mmol/L	50.4 % of 258 participants		55 % of 209 participants
Buckley et al.*,*2009	∗∗∗	Age >50, N = 1534; Male (46 %); Female (54 %) Primary & secondary prevention	History of MI/stroke/PAD/CHF or atrial fibrillation	Cross-sectional	Community	European Society of hypertension/European Society of Cardiology (ESH/ESC) 2007	<140/90 mmHg (non-diabetic) 130/80 mmHg (diabetic)			48.6 % of people without diabetes 16.7 % people with diabetes		
Kotseva et al.*,* 2009	∗∗	Age ≥18 & <80, N = 386; secondary prevention participants	Acute MI/Acute myocardial ischemia	Retrospective cohort	Hospital	Joint European Societies' guidelines (2003)	<140/90 mmHg (non-diabetic) 130/80 mmHg (diabetic)		<4.5 mmol/L	48 % of 354 people with and without diabetes.		75 % of 344 patients
Bermingham et al., 2011	∗∗∗	Age ≥55, N = 183; Male (45 %); Female (54.6 %) Primary and secondary prevention	Coronary artery disease/hypercholesterolemia/hypertension/or Arrythmia	Prospective Cohort	Community	European guidelines on cardiovascular disease prevention in clinical practice 2007		<2.5 mmol/L <3 mmol/L			Overall, 71.7 % of a total 669 participants who are prescribed statins attained their LDL-C goals based 10-year fatal CVD risk	
Dunne et al.*,* 2013	∗∗	Age >30, N = 488; Primary and secondary prevention	Coronary heart disease	Retrospective audit	Hospital; Outpatient clinic in academic teaching hospital	The National Cholesterol Education Program III	Systolic BP <130 mmHg Diastolic BP < 85 mmHg	<2.5 mmol/L in people with CHD <3 mmol/L in people without CHD		People with CHD (112 participants): 64 % achieved the systolic goal & 44 % achieved the diastolic goal People without CHD (376 participants): 36 % achieved the systolic goal & 56 % achieved the diastolic goal	42 % participants with CHD 43 % participants without CHD	
Murphy et al.*,* 2015	∗∗	Age >50, N = 3372; Male (45 %); Female (55 %) Primary and secondary prevention	Coronary heart disease	Cross-sectional	Community	Fourth (Fifth) Joint Task Force of the European Society of Cardiology and other societies on cardiovascular disease prevention in clinical practice ESC 2007 (ESC 2012)		<2.5 mmol/L according to the ESC 2007 <1.8 mmol/L according to the ESC 2012	<4.5 mmol/L according to the ESC 2007		**LDL-C 2.5 mmol/L:** 61 % of 166 known CVD 14.6 % (142) of people with 10-year fatal CVD risk score ≥5 % could achieve the goal **For LDL-C <1.8 mmol/L:** 27.7 % of 166 participants with known CVD	53 % of 166 participants with known CVD.
Gitt et al.*,* 2016	∗∗	Age≥ 45, N = 900; Primary and secondary prevention	Coronary heart disease/Peripheral artery disease	cross-sectional	Community & Hospital	European Society of Cardiology (ESC) and the European Atherosclerosis Society (EAS) Guidelines		<1.8 mmol/L (very high risk) <2.5 mmol/L (high risk) <3 mmol/L (non-high risk)			Overall, 43.5 % of total 900 participants, comprised of: 35.9 % of 618 very high risk participants. 58.6 % of 87 high risk participants. 64.4 % of 160 non-high risk participants.	
Kotseva *et, al* 2016	∗∗	Age ≥18 & <80, N = 201; Secondary prevention	Acute MI	Cross-sectional	Hospital; (Academic teaching hospital)	Joint European Societies guidelines (JES 2007 & JES 2012)	<130/80 mmHg <140/90 mmHg or 140/80 mmHg	<2.5 mmol/L <2 mmol/L <1.8 mmol/L		52.3 % participants attained the goal of BP < 130/80 mmHg 76.2 % participants attained the goal of BP < 140/90 or 140/80 mmHg	74.6 % participants attained the LDL-C goal of <2.5 mmol/L 47.5 % participants attained LDL-C goal of <2 mmol/L 34.8 % participants attained LDL-C goal of <1.8 mmol/L	
Ní Chróinín et al.,2018	∗∗∗	Age ≥18, N = 616; Male (50 %) Female (50 %) Secondary prevention	Ischemic stroke/transient ischemic attack	Prospective Cohort study	Community & Hospital	National Cholesterol Education Program (NCEP III)		<4.14 mmol/L (patients with 0–1 risk factors) <3.36 mmol/L (≥2 risk factors + 10-year risk ≤20 %) <2.59 mmol/L (≥2 risk factors + 10-year risk >20 %, or CHD/risk equivalent)			Overall, 54.1 % (200/370) achieved their recommended goals 80 % of 5 participants with (0–1) risk factors 66.4 % of 370 who have ≥2 risk factors + 10-year risk ≤20 % 46.2 % of 370 participants who have ≥2 risk factors + 10-year risk >20 %, or CHD/risk equivalent	
Cı'fkova et al., 2019	∗∗	Age >18 & < 80, N = 616; Secondary prevention	Coronary artery diseases	Cross-sectional	Hospital	European Society of Hypertension/European Society of Cardiology (EHS/ESC) 2013	**Systolic BP** <140 mmHg **Diastolic BP** <90 mmHg (non-diabetic) <85 mmHg (diabetic)			Overall, 73.9 % of 199 participants attained their BP goals 75.2 % of 157 men; 69 % of 42 women attained their BP goals		
Offiah et al.*,* 2022	∗∗	Age >50, N = 153; Male (68 %); Female (32 %) Primary and secondary prevention	Coronary artery disease/cerebrovascular disease (Stroke)/PAD	Cross-sectional	Hospital & Community; Academic teaching hospital	2016 & 2019 European Society of Cardiology/European Atherosclerosis Society (ESC/EAS) guidelines		**2016 Guidelines:** Low risk <3 mmol/L Moderate risk <3 mmol/L High risk <2.6 mmol/L, or 50 % reduction from the baseline Very high risk <1.8 mmol/L **2019 Guidelines:** Low risk <3 mmol/L Moderate risk <2.6 mmol/L High risk <1.8 mmol/L or ≥50 % reduction from the baseline Very high risk <1.4 mmol/L or ≥50 % reduction from the baseline			**According to 2016 guidelines:** Overall, 64.1 % of 153 participants 78.4 % of 74 participants treated for primary prevention 50.6 % of 79 participants treated for secondary prevention **According to the 2019 guidelines:** Overall, 40.5 % of 153 participants 60.8 % of 74 participants treated for primary prevention 21.5 % of 79 participants treated for secondary prevention	
McCaughey et al.*,* 2022	∗∗	Age >50, N = 163; Male (77 %); Female (23 %) Primary and secondary prevention	Atherosclerotic CVD	Retrospective review chart	Hospital	2016 & 2019 European Society of Cardiology guideline		**2016 Guidelines:** Low risk <3 mmol/L Moderate risk <3 mmol/L High risk <2.6 mmol/L, or 50 % reduction from the baseline Very high risk <1.8 mmol/L **2019 Guidelines:** Low risk <3 mmol/L Moderate risk <2.6 mmol/L High risk <1.8 mmol/L or ≥50 % reduction from the baseline Very high risk <1.4 mmol/L or ≥50 % reduction from the baseline			**According to 2016 guidelines:** 62 % of 99 very high risk participants 50 % of 2 high risk participants 73 % of 11 moderate risk participants **According to 2019 Guidelines:** 49 % of 43 very high risk participants 50 % of 2 high risk participants 60 % of 5 moderate risk participants.	

**Abbreviations:** CVD Cardiovascular disease; BP blood pressure; LDL-C Low density lipoprotein cholesterol; TC Total cholesterol; LLT Lipid-lowering therapy; TIA Transient ischemic attack; CHD Coronary heart disease; MI Myocardial infarction; PAD Peripheral artery disease. **As per The National Heart, Lung, and Blood Institute Quality Assessment Tool for Observational Cohort and Cross-sectional Studies** poor (∗); fair (∗∗); good (∗∗∗).

The BP and lipid targets applied varied across the included studies. For lipid targets; five studies applied LDL-C <3 mmol/L [5,6,28,29,33], seven studies had an LDL-C target of <2.5 or <2.6 mmol/L [5,6,28,29,31,33,35], and five studies applied a target of LDL-C <1.8 mmol/L [5,28,29,31,35]. Of the studies using LDL-C goal of <1.8 mmol/L, two studies conducted a comparative analysis between that goal and the stricter target of LDL-C < 1.4 mmol/L [5,29]. For BP targets; five studies applied <140/90 mmHg [11,12,30,31,34] and four studies applied intensive BP targets ≤130/80 or ≤130/85 [6,11,30,31] (Table 1).

**Fig. 2:**
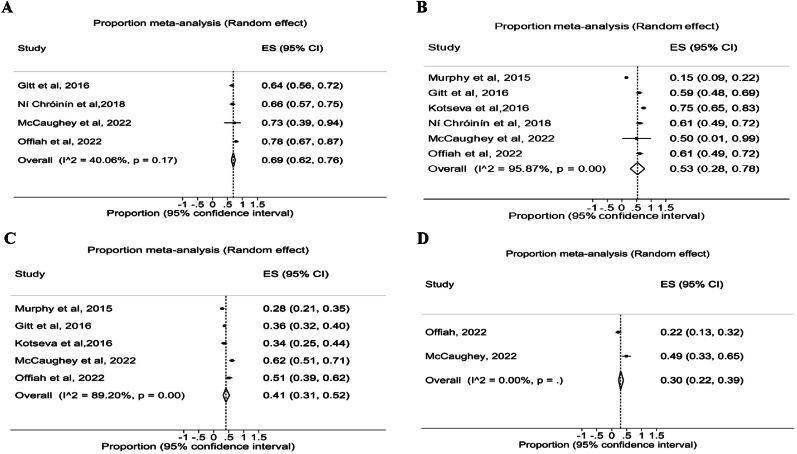
Meta-analysis for goal achievements using different LDL-C cutoff (A) LDL-C < 3 mmol/L; (B) LDL-C < 2.5 (2.6) mmol/L; (C) LDL-C < 1.8 mmol/L; (D) LDL-C < 1.4 mmol/L. Each black square represents the proportion of participants who attained LDL-C goals reported in each study and the extended lines represent the confidence intervals. **Abbreviations:** ES, Effect size; CI, Confidence interval.

The overall achievements of the LDL-C targets of <3 mmol/L, <2.5 mmo/L, <1.8 mmol/L, and <1.4 mmol/L in the pooled samples were 69 % ([95 % CI, 62, 76], I^2^ = 40.6 %), 53 % ([95 % CI, 28, 78], I^2^ = 95.87 %), 41 % ([95 % CI, 31, 52], I^2^ = 89.2 %) and 30 % ([95 % CI, 22, 39], I^2^ = 0 %) respectively (Fig. 2).

**Fig. 3:**
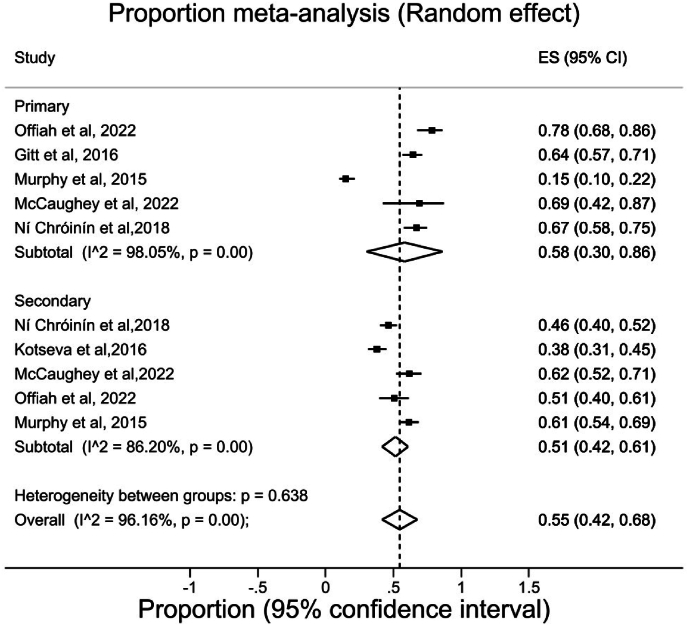
Meta-analysis of LDL-C goal attainment among participants treated for primary and secondary prevention. Each black square represents the proportion of participants who attained LDL-C goals reported in each study and the extended lines represent the confidence intervals. **Abbreviations:** ES, Effect size; CI, Confidence interval.

**Fig. 4:**
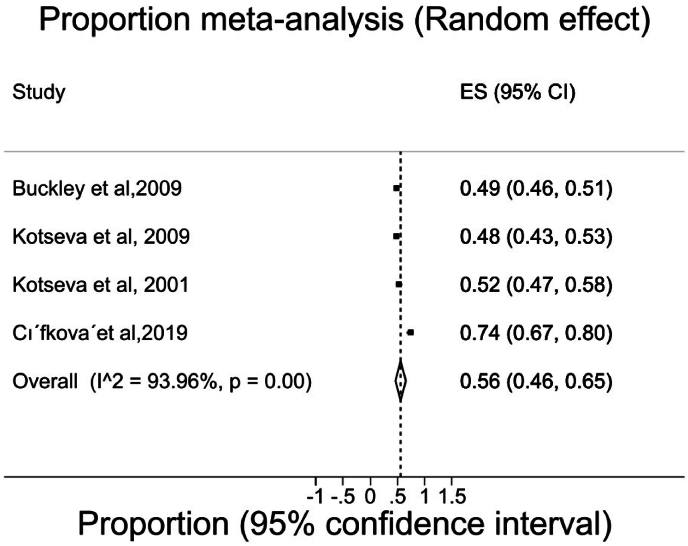
Meta-analysis of goal attainment for BP goal of <140/90 mmHg). Each black square represents the proportion of participants who attained BP goals of <140/90 mmHg reported in each study and the extended lines represent the confidence intervals. **Abbreviations:** ES, Effect size; CI, Confidence interval.

Of the pooled studies in the subgroup analysis, two studies were conducted in hospital settings [29,31], one study was conducted in community setting [35], and two studies were conducted in both community and hospital settings [28,33]. The overall proportion of participants treated for primary prevention who achieved their LDL-C target was 58 %, ([95 % CI, 30,86], I^2^ = 98.05 %), whilst the overall proportion of participants treated for secondary prevention who achieved target was slightly lower at 51 %, ([95 % CI, 42,61], I^2^ = 86.02 %) (Fig. 3),

In the pooled sample (random effect model), the overall proportions of people attaining the BP goal of <140/90 mmHg was 56 %, ([95 % CI 46 %,65 %]), I^2^ = 93.96 %). Of these studies, three studies were conducted in hospital settings [12,30,34] and one study was conducted in community setting where a relatively low proportion of people could meet the BP goal [11](Fig. 4).

### 3.2 Medication adherence

Nine studies reported the level of adherence to antihypertensives and LLT among people treated for primary or secondary CVDs prevention [36,[38], [39], [40], [41], [42], [43], [44], [45]]. Six studies were conducted in community settings [36,39,41,[43], [44], [45]] and three studies in hospital settings [38,40,42]. The participant mean age (± standard deviation) ranged from 64.9 (±9.9) [36] to 77 (±5.4) years [44]. Two studies included people treated for secondary CVDs prevention [38,40], one study included people treated for primary CVDs prevention [43], and six studies included people treated for both primary and secondary CVDs prevention [36,39,41,42,44,45]. The methods used to assess medication adherence in the included studies were self-report questionnaires, prescription refill data, or direct observation methods. Self-report questionnaires used were the 8-item Morisky Medication Adherence Scale (MMAS) [40,41], the 4-item MMAS [36], a self-reported assessment of antihypertensive medication adherence using a questionnaire based on the validated Brief Medication Questionnaire which includes patients self-reported adherence and barriers to adherence identified by patients [42], and a self-reported questionnaire in which participants answered questions either over the phone, paper survey, or through study follow-up [38,40]. Methods of measuring prescription refill data were proportion of days covered (PDC) [39,41,45], and medication possession ratio (MPR) [44]. Urine analysis using mass spectrometry was used as a direct method to assess medication adherence in two studies [42,43]. Studies are described in Table 2.

**Table 2:** Characteristics and results of the studies included for medication adherence (9 studies).

Assessment tools		Studies	Quality assessment	Participants demographics	Setting	Medications	Adherence levels
**Report** **Scale/Questionnaire**	**8-item Morisky Medication Adherence Scale**	Murphy et al., 2015	∗∗	Age ≥50, N = 103	Hospital	Secondary prevention medications	41.2 % of 103 participants were high adherent; 36.8 % were medium adherent; 12.2 % were low adherent.
		Dillon et al., 2018	∗∗∗	Age ≥70, N = 784	Community	Antihypertensive medications	51.5 % of 784 participants were high adherent; 36.2 % were medium adherent; 12.2 % were low adherent.
	**4-Item Morisky Medication Adherence Scale**	Bermingham et al.*,* 2011	∗∗∗	Age >55, N = 183	Community	Statin/LLT	51 % of 119 participants were adherent.
	**Self-reported adherence through phone calls/paper surveys/study follow-up**	Murphy et al.*,* 2015	∗∗	Age ≥50, N = 114;	Hospital	Secondary prevention medications	11.4 % of 114 participants missed their medications at least once in the preceding fortnight; 54 % never forget their medications.
						Antihypertensive medications adherence	89 % of 114 participants continued using medications until follow-up.
						Statin/LLT	87.7 % of 114 participants continued using medications until follow-up.
		Keating et al.,2022	∗∗	Age 47–86, N = 173	Hospital	Secondary prevention medications	93 % of 173 participants continued on using medications until follow-up.
		Curneen et al.,2022	∗∗∗	Age ≥18, N = 73	Hospital	Antihypertensive medications	27 % of 73 participants were adherent.
**Pharmacy** **Refill** **Records**	**Proportion of days covered (PDC)**	Dillon et al., 2018	∗∗∗	Age ≥70, N = 573	Community	Antihypertensive medications	9 % of 573 participants were non adherent.
		Dillon et al.,2019	∗∗∗	Age ≥60, N = 905	Community	Antihypertensive medications	92 % of 905 participants continued on using medications.
		Walsh et al.,2019	∗∗∗	Age 67–79, N = 1431	Community	Antihypertensive medications	72.9 % of 1431 participants continued on using medications.
	**Medication possession ratio (MPR)**	Kim et al., 2017	∗∗∗	Age >70, N = 855; n = 518 had hyperlipidemia n = 183 had hypertension	Community	Antihypertensive medications	28 % of 183 participants were non-adherent.
						Statin/LLT	29.5 % of 518 participants were non-adherent.
	**Group based trajectory modelling (GBTM)**	Dillon et al., 2019	∗∗∗	Age ≥60, N = 905	Community	Antihypertensive medications	58 % of 905 participants were very high adherent; 35.8 % were high adherent; 6 % were low adherent.
**Direct**	**Mass spectrometry (urine-analysis)**	Curneen et al., 2022	∗∗∗	Age ≥18, N = 73	Hospital	Antihypertensive medications	27 % of 73 participants were adherent.
		Hayes et al., 2019	∗∗	Age ≥60, N = 235	Community	Antihypertensive medications	74 % of 235 participants were high adherent; 23.8 % were medium adherent; 2 % were non-adherent.

Secondary prevention medications include antihypertensive medications and/or lipid lowering therapy**. As per The National Heart, Lung, and Blood Institute Quality Assessment Tool for Observational Cohort and Cross-sectional Studies** poor (∗); fair (∗∗); good (∗∗∗).

Seven studies reported the level of adherence to antihypertensive medications [[39], [40], [41], [42], [43], [44], [45]]. In Murphy et al., of 103 participants, 41 %, 37 % and 12 % reported high, medium, and low adherence respectively to secondary CVDs prevention medications including antihypertensive medications using 8-item Morisky Medication Adherence Scale [40]. Similarly, in Dillon et al., 2018, 52 %, 36 %, and 12 % were high, medium and low adherent to antihypertensive medications using the same assessment tool [41]. Using the PDC measure, 73 % of people prescribed antihypertensives were classified as adherent (PDC ≥80 %) in a study by Walsh et al. [39] and 92 % were classified as adherent by the same measure in Dillon et al. [45]. Dillon et al., further reported that 58 %, 36 %, and 6 % of participants had very high, high and low level of adherence to antihypertensive medication using group-based trajectory modelling. However, Curneen et al., 2022, reported that only 36.5 % of 55 participants who self-reported adherence to their antihypertensive medications were truly adherent based on their urine analysis results [42]. Using the direct assessment tool of urine analysis, Hayes et al. found that 74 % of a total 235 participants with resistant hypertension were fully adherent [43]. Medication non-adherence was assessed in two studies using PDC threshold of 0.8 and MPR threshold of 0.8 and it was found that 9 % and 28.4 % were non-adherent to antihypertensive medications respectively [41,44].

Two studies reported the adherence to lipid lowering medications; Murphy et al. reported that 87.7 % of the included participants self-reported adherence to statin therapy during the study follow-up [40]. Bermingham et al. reported non-adherence to statins was 48.7 % using the self-report 4-item MMAS, where 72 % of those reporting non-adherence were unintentionally non-adherent and 25.9 % were intentionally non-adherent [36].

Patient factors associated with antihypertensive medications adherence were reported to be using single pill combination, age (≥75 years) [39], beliefs that secondary prevention medications including antihypertensive medications and LLT will protect from further stroke events [40] and admission to the hospital [40]. However, in Hayes et al. it was reported that there was no statistically significant difference in rate of antihypertensive medication adherence by gender [43].

### 3.3 Guideline adherence

Six studies included data regarding adherence to guideline recommendations among people receiving primary and secondary antihypertensives and LLT [11,[33], [34], [35],^46^,^47^] (Table 3). The number of the included participants ranged from 134 to 28,683 [46,47] respectively, and their mean age (± standard deviation) ranged from 64.7 (±11.9) [11] to 71.3 (±13) years [33]. Three studies documented the prescribed medications among people post-acute events including acute myocardial infarction (MI) [34,47] and stroke [33]. According to the EuroAspire II report, 70.4 % of the 297 people who should be on LLT actually are; however, among people on LLT, 45 % still had TC levels which were above the recommended goal of 5 mmol/L [34]. In a national study that included 134 people post-MI, 87 % were prescribed statin therapy on hospital discharge [47]. Murphy et al. examined the statin utilization among participants with high risk CVDs including patients with known CVDs and found that only 67 % of 166 patients were using statin treatment, despite the guideline recommending statins for everyone with CVDs. The level of statin utilization varied based on type of CVDs event, with 80.3 % of people who reported previous revascularization taking statins compared to only 51.6 % of people who self-reported transient ischemic attacks (TIA) (51.6 %). In addition, only 57.4 % of 141 patients with diabetes were utilizing statin, despite the guideline recommending statin use in all with diabetes [35]. Similarly, 24 % of people post stroke/TIA were reported not to be taking a statin and 15 % of 196 people with LDL-C above the normal levels (LDL-C >2.59 mmol/L) were not on any LLT in another study [33]. The trend of antihypertensive medication prescribing in a primary care setting was examined in relation to the British Hypertension Society guidelines by Okechukwu et al. who reported greater adherence to the clinical guideline recommendations among participants aged <55 years than participants aged ≥55 years (OR: 1.31 95 % CI 1.26, 1.37). Antihypertensive choice did not always match with the guideline recommendations, for example, angiotensin converting enzyme inhibitors (ACEIs) were commonly prescribed to the participants aged ≥55 years (26.9 %) and beta-blockers were often prescribed to participants aged <55 years (36 %) [46]. Buckley et al., reported guideline adherence by physicians to the British Hypertension Society Guideline was 54 %, and to the ESH/ESC guideline was 38 % [11]. However, 57.5 % of the participants did not receive any further action such as increasing the dose, switching to a new medication or combination therapy to control their BP as their physicians considered their BP on target, even though it was not within the clinical guideline goals [11].

**Table 3:** Characteristics and results of studies reporting guideline adherence (6 studies).

Author, Year	Quality assessment	Design	Setting	Demographics	Type of Population	Guideline	Guideline recommendation	Guideline adherence
Kotseva, 2007	∗∗	Cross-sectional	Hospital	Age ≤70 N = 339	Secondary prevention (MI)	Second Joint European Societies Task Force 1998	• TC should stay below 5 mmol/L and LDL-C should be below 3 mmol/L • The majority of patients with coronary or atherosclerotic diseases need lipid lowering therapies (LLT)	**For people with TC ≥ 5 mmol/L** • 26 % not on LLT; 27.7 % on LLT **Among people with history of hyperlipidemia** • 15.4 % of 247 not on LLT; of them 73.7 % had TC ≥ 5 mmol/L. • 84.6 % of 247 on LTT; only 55 % achieved the goal of TC < 5 mmol/L.
Syed et al.*, 2010*	∗∗	Retrospective analysis of cases	Hospital	N = 134	Secondary Prevention (Post MI)	AHA/ACC Guidelines for Secondary Prevention of Patients with Coronary and other Atherosclerotic Vascular Disease 2006.	• Patients with LDL-C 100 mg/dL (2.6 mmol/L) or above should be prescribed LLT on hospital discharge, with preference given to statins	• Out of 134 participants discharged after MI, 116 (87 %) were prescribed statins on discharge.
Murphy, 2015	∗∗	Cross-sectional	Community	Age >50, N = 3372; Male (44.9 %) Female (55 %)	Primary & secondary prevention	Fourth Joint Task Force of the European Society of Cardiology and other societies on cardiovascular disease prevention in clinical practice (ESC 2007)	• Statin therapy is recommended for all CVD and most diabetic patients. • If total CVD risk ≥5 %, lipid-lowering drug therapy should be considered. • In all patients with an acute coronary syndrome, statin treatment should be initiated while the patients are in the hospital	**Statin utilization was found as below:** • 68.6 % of the participants with known CVD. • 57.4 % of the participants with diabetes. • 19.7 % of participants with 10-year fatal CVD risk score ≥5 % • 78.6 % of the participants who had experienced CV events
Ní Chróinín et al.*, 2018*	∗∗∗	Prospective cohort study	Community & Hospital	Age >60 N = 616 Male (49.8 %) Female (50 %)	Secondary Prevention (Ischemic stroke/TIA)	National Cholesterol Education Program (NCEP III)	• If CHD/risk equivalent, or 2+ risk factors & 10- year risk (FRS) > 20 %: start LLD if LDL ≥130 mg/dl; consider LLD if LDL 100–129 mg/dl • If 2+ risk factors & 10-year risk consider LLD if consider LLD if LDL ≥160 mg/dl • If 0–1 risk factor: consider LLD if LDL>190 mg/dl • LDL measurement following stroke and TIA and pre-discharge initiations of statins	**Statin utilization prior to an event:** • 48.5 % of 298 patients with CHD or risk equivalent. • 21.6 % of 245 patients who met the NCEP III criteria for LLT, were not on treatment. **After stroke/TIA:** • 24 % of patients were not on any pharmacological treatment. **Of patients with high LDL-(C>2.59 mmol/L)** • 15 % of 196 were not prescribed statin therapy.
Okechukwu et al.*, 2007*	∗∗	Retrospective Cohort	Community	Under 55 years: 12,745 55 years and over: 28,683 Male (39 %); Female (60.7 %)	Primary Prevention	British Hypertension Society guidelines 2004	**Initiation of antihypertensive medications as the following:** • ACEIs/ARBs or β-blockers in patients under 55 years old. • Calcium channel blockers (CCBs) or diuretics for patients 55 years and over. • Beta blockers should be used with caution for diabetic patients	**For people aged under 55 years without anti-diabetic medications:** • 22.5 % were prescribed ACEIs. • 36 % were prescribed β-blockers. • 15 % were prescribed CCBs. • 26 % were prescribed diuretics. **For people aged 55 years and over without anti-diabetic medications:** • 19 % were prescribed CCBs. •32.7 % were prescribed diuretics. • 26.9 % were prescribed ACEIs. • 21.3 % were prescribed β-blockers. **For people with diabetes:** • 10.5 % were prescribed β-blockers. • 46.7 % were prescribed ACEIs • 17 % were prescribed CCBs • 25.6 % were prescribed diuretics
Buckley et al.*, 2009*	∗∗∗	Cross-sectional	Community	Age >50 N = 1534 Male (46 %); Female (53.8 %)	Primary & Secondary Prevention	European Society of Hypertension/European Society of Cardiology ESH/ESC 2007	• ESH/ESC 2007 recommended escalation to combination therapy after full dose monotherapy has failed. • Combination therapy is recommended for first-line treatment for patients with systolic BP > 160 mmHg or diastolic BP > 100 mmHg, and/or high and very high risk patients.	**Antihypertensive monotherapy prescribing**: • 40.5 % participants were prescribed monotherapy • Of them, 32 % were prescribed ACEIs, 22 % were prescribed ARBs, 39.8 % were prescribed β-blockers, 29.7 % were prescribed diuretics, 27.9 % were prescribed CCBs. **Combination therapy:** • 6.6 % & 8 % used ACEIs or ARBs with diuretics. • 1.2 % were using ACEIs with CCBs.

**Abbreviations** TC Total Cholesterol; LDL-C low density lipoprotein; LLT lipid lowering therapy; ACEIs angiotensin converting enzyme inhibitors; ARBs angiotensin receptor blockers; CCBs calcium channel blockers; ESH/ESC European Society of hypertension/European Society of Cardiology; MI myocardial infarction; TIA transient ischemic attack. **As per The National Heart, Lung, and Blood Institute Quality Assessment Tool for Observational Cohort and Cross-sectional Studies** poor (∗); fair (∗∗); good (∗∗∗).

## 4 Discussion

This systematic review is the first to assess the achievement of BP and lipid clinical guideline targets, medication adherence among patients and guidelines adherence among prescribers across studies conducted in Ireland. The results showed that LDL-C goal attainment is low especially when stringent LDL-C cutoff levels is recommended including LDL-C <2.5 mmol/L and LDL-C <1.8 mmol/L as the proportions were 53 % (28,78) and 41 % (31,52) respectively. In addition, antihypertensive choice did not always match the guideline recommendations; 15%–27.9 % were prescribed calcium channel blockers and 21.3%–39.8 % were prescribed β blockers, both of which are not first-line antihypertensives in many situations [11,46]. LLT was not prescribed to many patients whose risk of CVD warranted LLT, including 27.7 % of people with TC > 5 mmol/L and 48.5–68.6 % of people with known CVD [[33], [34], [35]]. Medication adherence varied according to the assessment tool used. In Ireland, cardiovascular diseases kill around 9000 people every year. These deaths still occur, despite the finding that 80 % of premature CVDs are preventable [48]. Stamenic et al., reported that individuals with CVDs seek healthcare services to a greater extent than individuals without CVDs [49]. In 2022, the cost of healthcare services use associated with CVDs among Irish adults aged >50 was €352 million, with hospital admissions accounting for 80 % of this cost [49]. Therefore, the preventive approach to CVDs is crucial to protect people from developing CVDs and to reduce the risk of repeat cardiovascular events.

It is difficult to estimate the total number of people being treated with lipid-lowering therapy or antihypertensives in Ireland. However, data produced by the Primary Care Reimbursement Service (PCRS) in Ireland, shows that statins atorvastatin and rosuvastatin are consistently in the top 10 dispensed medicines across PCRS schemes. Also, antihypertensives such as bisoprolol, ramipril and amlodipine all feature in the top 20 dispensed medicines across PCRS schemes. This demonstrates the prevalence with which community-dwelling adults are taking such medicines, whether for primary or secondary prevention [50].

The review findings showed that there is variation in the achievement of LDL-C and BP targets. The proportion of primary prevention participants who attained LDL-C goals was higher than the proportion of secondary prevention participants achieving this goal. However, primary prevention participants were categorized as low risk or moderate risk patients; therefore, the less stringent LDL-C goal of <3 mmol/L was applied in the majority of included studies. On the contrary, the LDL-C goal of <2.5 mmol/L and <1.8 mmol/L were used for high risk and very high risk participants who constituted the secondary prevention group [51]. This aligns with the findings of a review of European settings, which reported that the pooled estimate for goal achievement was higher among high risk patients with LDL-C target of <2.5 mmol/L compared to very high risk patients with LDL-C goal of <1.8 mmol/L, with 46 % vs 18 % achieving their LDL-C goal, respectively [52]. The more aggressive LDL-C target of <1.4 mmol/L recommended in the 2019 ESC guidelines [53] was achieved by an even lower proportion of very high risk population with a pooled estimate of 28 %. A retrospective observational cohort study conducted in Wales reported that only 23.4 % of 2353 participants achieved 2019 ESC goals of LDL-C <1.4 mmol/L, one year post percutaneous coronary intervention; however, 47.8 % of 4812 participants achieved the LDL-C <1.8 mmol/L [54].

In the present review there is a discrepancy in the proportion of participants who achieved the BP goal of <140/90 mmHg (56 %) and participants who achieved the stringent BP goal of <130/80 mmHg (37 %). In this systematic review, only Buckley et al., 2019 reported proportion of people who achieved BP goals in community settings [11] which was lower than the proportion of people who achieved goals in hospital settings [6,12]. These findings are broadly similar to studies carried out in other countries. A previous narrative review that included 12 studies evaluating the level of BP goal attainment among participants with hypertension, diabetes or stroke reported that the proportion of participants who attained BP goals ranged from 25 % to 76.4 % [55]. According to Vickie Andros et al., who reported the level of BP control among patients with diabetes in the United States; only 31.4 % of 1328 participants achieved BP control of <130/80 mmHg [56,57]. Similarly Barrios et al., examined BP goal achievement in a primary care setting in Spain, where the proportion of participants who attained the BP goal of <140/90 mm Hg (or < 130/80 mm Hg for diabetic) was 24.8 % [58]. In a single center, cross-sectional study of hypertensive outpatients in Italy, 36.6 % of 1578 participants treated with antihypertensive mono-therapy achieved BP goals of <140/90 mm Hg [59]. Unlike the PORTRAIT-DYS study which found that women were 22 % less likely to achieve their LDL-C goal than men, the studies in this review did not identify any patient or prescriber factors to be associated with goal attainment [60].

Medication adherence can reduce healthcare utilization and healthcare costs. Dillon et al., 2019, reported that each 10 % increase in the refill adherence to antihypertensive medications was associated with a 16 % lower rate of outpatient hospital visits in Ireland [45]. In a large retrospective study conducted in the United States; healthcare costs were reduced by $387-$813 for patients adherent to antihypertensive medications, compared to those who are non-adherent [61]. This systematic review identified heterogenous methodologies reporting medication adherence among participants prescribed antihypertensive medications and lipid lowering medications. The proportion of participants who were adherent to antihypertensive medications ranged from 27 % to 92 % [42,45]. Comparable findings were in a retrospective analysis of antihypertensive medications adherence in the U.S which reported that among 625,620 participants, 15.4 % were moderately adherent (MPR, 60%–79 %), and 74.6 % of the participants were reported high adherent to antihypertensive medications using the MPR of >80 % as the threshold [61]. Similar to the findings from Walsh et al. [39], Beall et al. have reported that age is significantly associated with increasing medication adherence; participants aged 65 years and older were more persistent to their antihypertensive medications (54.6 %) compared to those aged 18–44 years (39 %) [62].

Guideline adherence by clinicians also contributes to improved outcomes in patients with cardiovascular diseases [63]. Whilst clinicians are aware that clinical guidelines are helpful educational resources and are intended to improve the quality of care provided to patients, they considered them impractical and too rigid to apply in a person-centered manner [64]. In Buckley et al., 54 % of physicians in primary care were adherent to the British Hypertension Society Guideline and 38 % were adherent to the ESH/ESC guidelines [11]. Similarly, a cross-sectional study examining physicians' adherence to hypertension guidelines when prescribing antihypertensive medications for hospitalized patients in an Indian tertiary care hospital reported that out of 198 physicians, only 51 % of the physicians were adherent to The Seventh Report of the Joint National Committee on Prevention, Detection, Evaluation, and Treatment of High Blood Pressure (JNC7), 59 % were adherent to JNC8, and 31 % were adherent to The 2017 American College of Cardiology/American Heart Association guidelines [65]. The identified studies do not show full compliance with the guideline recommendations. Despite the solid evidence on the benefits of using LLT especially among people who are treated for secondary prevention, a gap exists between guideline recommendations and the clinical practice [[66], [67], [68]]. Syed et al., and Murphy et al., reported that not all people who were discharged post MI or with known CVDs were prescribed LLT [35,47]. On the other hand, Kotseva et al. reported that more than half of the people who were on LLT still have TC level above 5 mmol/L [34]. Although the pattern of choosing the initial antihypertensive medications was relatively similar to studies in Europe and UK as reported in Okechukwu's study [46,69,70], Buckley et al. reported that prescribers miss necessary escalation of antihypertensive monotherapy into combination therapy.

There are limited data about guideline adherence, and in particular its relationship with goal achievements, in Ireland. Another study described the LLT pattern among German people in general practices where 2.8 % were using high-intensity statin, 32 % were using low-moderate intensity statin, and 1.3 % were on non-statin therapy. Among patients with atherosclerotic cardiovascular diseases (recent acute coronary syndrome, ischemic stroke, or peripheral artery disease), 44.9 % received any LLT and 43.6 % of patients received prescriptions for statins on the index date. However, 8.5 % of patients achieved LDL-C <1.8 mmol/L and 25.6 % of the participants achieved LDL-C 1.8 to <2.6 mmol/L [20]. These European studies have shown that secondary prevention of CVDs is often undertreated, and often the target goals set out by the guidelines are not achieved.

Although none of the included studies assessed the relationship between achieving LDL-C goals and prescriber adherence to guideline recommendations and/or patient adherence to medications within Irish context, Parris et al., reported a significant correlation between the medication possession ratio (MPR) for statin therapy and LDL-C goal achievements among people with dyslipidemia and diabetes [71]. Similarly, a Norwegian study reported a strong association between the use of LLTs for secondary prevention and the achievement of LDL-C goals, but the same study did not find an association between using antihypertensives and achieving target BP. However, the study did not assess the level of medication adherence among participants and the sample size may have been underpowered for hypertensive patients [19]. However, Bramley et al., reported that people with high adherence to antihypertensive medications were 45 % more likely to achieve the BP goals compared to medium and low adherence with monotherapy after reviewing the medical and pharmacy claims from 13 U S. health plans [21]. On the other hand, Vashitz et al., assessed the rates of physician adherence to prescribing guidelines and found low rates of adherence to guidelines for both LLT initiation and up-titration, but found relatively high rates of medication adherence by patients [72]. This review has found studies which independently examine goal attainment in CVD, prescriber adherence to guidelines and medication adherence by patients. However, there are a lack of studies examining the relationship between guideline adherence, medication adherence and goal attainment in CVDs.

As with all systematic reviews, many of the limitations of the review reflect those of the included studies. The quality of the included studies was assessed using an established tool. The quality of some included studies was scored fair; this was primarily due to the nature of the cross-sectional studies, namely having no time lag between the exposure and the outcome, and low sample size in some studies. However, funnel plot asymmetry was not conducted as few studies (<10 unique studies) were included in each meta-analysis [73].

None of the included studies assessed the relationship between medication adherence or guideline adherence and the achievement of BP or LDL-C goals. A large number of studies were conducted in hospital settings rather than community settings; that might lead to higher values of pooled estimates, as the patients were being monitored. All meta-analyses in this review showed a statistically significant heterogeneity. As this was a proportional meta-analysis it is expected that the I^2^ test for prevalence pooled estimates is high due to the difference in the time and place where the included studies were conducted [27]. The heterogeneity in our review resulted from the variation in BP and LDL-C targets across the included studies and the variation in whether the participants were treated for primary or secondary CVDs prevention. Although some studies recruited participants from hospital and community settings, there were not enough findings to examine the differences in goal achievements in both settings. In addition, there are few studies that examine the differences in goal achievements and medication adherence among people treated for primary and secondary prevention.

## 5 Conclusion

This review has found evidence that prescriber adherence to guidelines for the treatment of CVDs is low, patients’ adherence to medications for CVDs is variable, and that the achievement of LDL-C and BP goals is often concerningly low. Nationally representative studies examining objective measures of medication adherence, guideline adherence and goal attainment are needed to examine the relationship between these three variables in CVDs, as CVDs remain a leading cause of morbidity and mortality across the globe.

## CRediT authorship contribution statement

**Rehab Elhiny:** Writing – original draft, Visualization, Investigation. **Linda M. O'Keeffe:** Supervision, Methodology. **Elizabeth O. Bodunde:** Validation. **Stephen Byrne:** Writing – review & editing, Supervision. **Maria Donovan:** Writing – review & editing, Supervision. **Margaret Bermingham:** Writing – review & editing, Supervision, Conceptualization.

## Data availability

The data underlying this article will be shared on reasonable request with the corresponding author.

## Declaration of competing interest

The authors declared no conflict of interest.
