# Supplementary material for: Goal attainment, medication adherence and guideline adherence in the treatment of hypertension and dyslipidemia in Irish populations: A systematic review and meta-analysis
Source: Int J Cardiol Cardiovasc Risk Prev. 2025 Jan 4;24:200364. doi: 10.1016/j.ijcrp.2025.200364 (PMC11773485; doi:10.1016/j.ijcrp.2025.200364)
Supplement: Multimedia component 1 [file mmc1.docx]

**Supplementary Table 1: Search strategy adopted for each electronic database.**

| Data base | Search strategy |
| --- | --- |
| PubMed | (("cardiovascular diseases"[MeSH Terms] OR "dyslipidemias"[MeSH Terms] OR "cerebrovascular disorders"[MeSH Terms] OR "lipoproteins"[MeSH Terms] OR "cardiovascular disease*"[Title/Abstract] OR "Heart disease"[Title/Abstract] OR "cerebrovascular disease*"[Title/Abstract] OR "Coronary heart disease"[Title/Abstract] OR "Coronary artery disease"[Title/Abstract] OR "Ischemic Heart disease"[Title/Abstract] OR "CAD"[Title/Abstract] OR "CHD"[Title/Abstract] OR "CVD"[Title/Abstract] OR "IHD"[Title/Abstract] OR "Stroke"[Title/Abstract] OR "Atherosclerosis"[Title/Abstract] OR "HTN"[Title/Abstract] OR "hypertens*"[Title/Abstract] OR "High blood pressure"[Title/Abstract] OR "Systolic"[Title/Abstract] OR "Diastolic"[Title/Abstract] OR "Blood pressure"[Title/Abstract] OR "DBP"[Title/Abstract] OR "SBP"[Title/Abstract] OR "BP"[Title/Abstract] OR "Dyslipidemia"[Title/Abstract] OR "Hypercholesterolemia"[Title/Abstract] OR "Hypercholesteremia"[Title/Abstract] OR "Hyperlipidemia"[Title/Abstract] OR "Cholesterol"[Title/Abstract] OR "Lipoprotein"[Title/Abstract] OR "LDL"[Title/Abstract] OR "LDL-Cholesterol"[Title/Abstract] OR "triglyceride*"[Title/Abstract] OR "lipid*"[Title/Abstract] OR "HDL"[Title/Abstract] OR "Low density lipoprotein"[Title/Abstract] OR "Hypertriglyceridemia"[Title/Abstract] OR "Apolipoprotein B"[Title/Abstract] OR "Apo B"[Title/Abstract]) AND (("patient compliance"[MeSH Terms] OR "Guideline adherence"[MeSH Terms] OR "Patients compliance"[Title/Abstract] OR "Medication adherence"[Title/Abstract] OR "Patient nonadherence"[Title/Abstract] OR "Patients non-adherence"[Title/Abstract] OR "Persistence"[Title/Abstract] OR "Adherence"[Title/Abstract] OR "Concordance"[Title/Abstract] OR "Compliance"[Title/Abstract] OR "Patient noncompliance"[Title/Abstract] OR "Medication nonadherence"[Title/Abstract] OR "Guideline adherence"[Title/Abstract] OR "Guideline Compliance"[Title/Abstract] OR "Guideline implementation"[Title/Abstract] OR "Guideline nonadherence"[Title/Abstract] OR "Guideline directed medical therapies"[Title/Abstract] OR "GDMT"[Title/Abstract]) OR (((((("Goal* achieve*"[Title/Abstract]) OR ("Target* achieve*"[Title/Abstract])) OR ("Target* attain*"[Title/Abstract])) OR ("Goal* attain*"[Title/Abstract])) OR ("Therapeutic goal*"[Title/Abstract])) OR ("Therapeutic target*"[Title/Abstract])))) AND (((((((((((Ireland[Title/Abstract]) OR ("Republic of Ireland"[Title/Abstract])) OR (Irish[Title/Abstract])) OR ("Northern Ireland"[Title/Abstract])) OR (ireland, republic of[MeSH Terms])) OR (ireland[MeSH Terms])) OR (northern ireland[MeSH Terms])) OR Ireland[Affiliation])) OR ("Republic of Ireland"[Affiliation])) OR (Irish[Affiliation])) OR ("Northern Ireland"[Affiliation])) |
|  | ((TITLE-ABS-KEY("Cardiovascular disease*" OR "Heart disease*" OR "Cerebrovascular disease*" OR "Cerebrovascular disorder*" OR "Coronary heart disease" OR "Coronary artery disease" OR "Ischemic heart disease" OR "CAD" OR "CVD" OR "CHD" OR "IHD" OR "Stroke" OR "Atherosclerosis")) OR (TITLE-ABS-KEY("HTN" OR "Hypertens*" OR "High blood pressure" OR "Systolic" OR "Blood pressure" OR "DBP" OR "SBP" OR "BP" OR "Diastolic")) OR (TITLE-ABS-KEY("Dyslipidemia*" OR "Hypercholesteremia" OR "Hypercholesterolemia" OR "Hyperlipidemia" OR "Cholesterol" OR "Lipoprotein*" OR "LDL" OR "ldl-cholesterol" OR "Triglyceride*" OR "Lipid*" OR "HDL" OR "Low density lipoprotein" OR "Hypertriglyceridemia" OR "apolipoprotein B" OR "Apo-B"))) AND (((TITLE-ABS-KEY("Patients Compliance" OR "Patient Compliance" OR "Medication adherence" OR "Patient nonadherence" OR "patients non-adherence" OR "Persistence" OR "Patient constancy" OR "Adherence" OR "Concordance" OR "compliance" OR "Patient noncompliance" OR "Medication nonadherence")) OR (TITLE-ABS-KEY("Guideline adherence" OR "Guideline compliance" OR "Guideline implementation" OR "Underutilization evidence-based therapies" OR "Guideline nonadherence" OR "Guideline directed medical therapies" OR "GDMT"))) OR ((TITLE-ABS-KEY("Target* achieve*") OR TITLE-ABS-KEY("Target* attain*") OR TITLE-ABS-KEY("Goal* achieve*") OR TITLE-ABS-KEY("Goal* attain*") OR TITLE-ABS-KEY("Therapeutic goal*")))) AND ((TITLE-ABS-KEY(Ireland) OR TITLE-ABS-KEY("Republic of Ireland") OR TITLE-ABS-KEY(Irish) OR TITLE-ABS-KEY("Northern Ireland"))) OR (AFFIL(Ireland OR Irish OR "Republic of Ireland" OR "Northern Ireland")) |
| Scopus |  |
| CINAHL | AB "cardiovascular disease*" OR AB "Heart disease*" OR AB "Cerebrovascular disease*" OR AB "Cerebrovascular disorder" OR AB "Coronary heart disease" OR AB "Coronary artery disease" OR AB ischemic heart disease" OR AB CAD OR IHD OR AB CVD OR AB CHD OR AB Stroke OR Atherosclerosis OR AB HTN OR AB Hypertens* OR AB "high blood pressure" OR AB Systolic OR AB Diastolic OR AB blood pressure OR AB DBP OR AB SBP OR AB BP OR AB dyslipidemia OR AB hypercholesteremia OR AB hypercholesterolemia OR AB hyperlipidemia OR AB cholesterol OR AB Lipoprotein* OR AB ldl OR AB ldl cholesterol OR AB ( Triglyceride* OR Hypertriglyceridemia ) OR AB Lipid* OR AB HDL OR AB ( low density lipoprotein OR "Apolipoprotein B" OR "Apo-B" ) AND AB "Patients compliance" OR AB "Patient compliance" OR AB "Medication adherence" OR AB "Patient nonadherence" OR AB "patients non-adherence" OR AB Persistence OR AB "Patient constancy" OR AB Adherence OR AB concordance OR AB Compliance OR AB "Patient noncompliance" OR AB "Medication nonadherence" OR AB "Guideline adherence" OR AB "Guideline compliance" OR AB "Guideline implementation" OR AB "Underutilization evidence-based therapies" OR AB "Guideline nonadherence" OR AB "guideline directed medical therapy" OR AB GDMT OR AB "Target*achieve*" OR AB "Goal*achieve*" OR AB "Goal*attain*" OR AB "Therapeutic goal" AND AB ireland OR AB irish OR AB republic of ireland OR AB northern ireland OR AF ireland OR AF irish OR AF republic of ireland OR AF northern ireland |
| Web of Science | TS=(“Cardiovascular disease*” OR “Heart disease*” OR “Cerebrovascular disease*” OR “Cerebrovascular disorder*”OR “Coronary heart disease” OR “Coronary artery disease” OR “Ischemic heart disease” OR “CAD” OR “CVD” OR “CHD” OR“IHD”OR “Stroke” OR “Atherosclerosis”) OR TS=(“HTN”OR “Hypertens*” OR “High blood pressure”OR “Systolic”OR “Blood pressure” OR “DBP”OR “SBP” OR “BP” OR “Diastolic”) OR TS=(“Dyslipidemia*” OR “Hypercholesteremia” OR “Hypercholesterolemia” OR “Hyperlipidemia” OR “Cholesterol” OR “Lipoprotein*” OR “LDL” OR “ldl-cholesterol” OR “Triglyceride*” OR “Lipid*” OR “HDL” “Low density lipoprotein” OR “Hypertriglyceridemia” OR “apolipoprotein B”OR “Apo-B” ) AND ((TS=("Patients Compliance" OR “Patient Compliance” OR “Medication adherence” OR “Patient nonadherence” OR “patients non-adherence” OR “Persistence” OR “Patient constancy” “Adherence” OR “Concordance” OR “compliance” OR “Patient noncompliance” OR “Medication nonadherence”)) OR TS=(“Guideline adherence” OR “Guideline compliance” OR “Guideline implementation” OR “Underutilization evidence-based therapies” OR “Guideline nonadherence” “Guideline directed medical therapies” OR “GDMT”)) OR TS=(“Target*achieve*”OR “Goal*achieve*” “Target* attain*”OR “Goal* attain*” OR “Therapeutic goal”) AND TS=(“Ireland” OR “Republic of Ireland” OR “Irish” OR “Northern Ireland”) |
| Embase | ('cardiovascular disease*':ti,ab,kw OR 'heart disease*':ab,ti OR 'cerebrovascular disease*':ab,ti OR 'cerebrovascular disorder':ab,ti OR 'coronary heart disease':ab,ti OR 'coronary artery disease':ab,ti OR 'ischemic heart disease':ab,ti OR cad:ab,ti OR cvd:ab,ti OR chd:ab,ti OR ihd:ab,ti OR stroke:ab,ti OR atherosclerosis:ab,ti OR htn OR hypertens*:ab,ti OR 'high blood pressure':ab,ti OR systolic:ab,ti OR 'blood pressure':ab,ti OR dbp:ab,ti OR sbp:ab,ti OR bp:ab,ti OR diastolic:ab,ti OR dyslipidemia*:ab,ti OR hypercholesterolemia:ab,ti OR hypercholesteremia:ab,ti OR hyperlipidemia:ab,ti OR cholesterol:ab,ti OR lipoprotein*:ab,ti OR ldl:ab,ti OR 'low density lipoprotein':ab,ti OR hypertriglyceridemia:ab,ti OR 'apolipoprotein b':ab,ti OR 'apo-b':ab,ti OR 'ldl-cholesterol':ab,ti OR triglyceride*:ab,ti OR lipid*:ab,ti OR hdl:ab,ti) AND ('patients compliance':ab,ti OR 'patient compliance':ab,ti OR 'medication adherence':ab,ti OR 'patient nonadherence':ab,ti OR 'patients non-adherence':ab,ti OR persistence:ab,ti OR 'patient constancy':ab,ti OR adherence:ab,ti OR concordance:ab,ti OR compliance:ab,ti OR 'patient noncompliance':ab,ti OR 'medication nonadherence':ab,ti OR 'guideline adherence':ab,ti OR 'guideline compliance':ab,ti OR 'guideline implementation':ab,ti OR 'underutilization evidence-based therapies':ab,ti OR 'guideline nonadherence':ab,ti OR 'guideline directed medical therapies':ab,ti OR gdmt:ab,ti OR 'target* achieve*':ti,ab,kw OR 'target* attain*':ti,ab,kw OR 'goal* achieve*':ti,ab,kw OR 'goal* attain*':ti,ab,kw OR 'therapeutic goal*':ti,ab,kw) AND (ireland:ab,ti OR 'republic of ireland':ab,ti OR irish:ab,ti OR 'northern ireland':ab,ti OR ireland:ff OR 'republic of ireland':ff OR irish:ff OR 'northern ireland':ff) |

**Supplementary Table 2: The National Heart, Lung, and Blood Institute Quality Assessment Tool for Observational Cohort and Cross-sectional Studies**

| **Study** | **1** | **2** | **3** | **4** | **5** | **6** | **7** | **8** | **9** | **10** | **11** | **12** | **13** | **14** | **Quality Rating** |
| --- | --- | --- | --- | --- | --- | --- | --- | --- | --- | --- | --- | --- | --- | --- | --- |
| The Davinci,2022 (1) | Yes | yes | Yes | Yes | Yes | Yes | No | Yes | Yes | No | Yes | NR | NA | No | Fair (9) |
| M. Dunne,2013 (2) | Yes | Yes | NR | Yes | No | Yes | Yes | Yes | Yes | No | Yes | NR | NR | No | Fair (8) |
| Peter Hayes, 2019 (3) | Yes | yes | yes | yes | Yes | Yes | No | yes | NR | No | yes | NA | NA | Yes | Fair (9) |
| Paul Dillon,2018(4) | Yes | Yes | Yes | Yes | Yes | Yes | Yes | Yes | Yes | Yes | Yes | CD | No | Yes | Good (12) |
| San Kim, 2017(5) | Yes | Yes | Yes | Yes | Yes | Yes | Yes | Yes | Yes | Yes | yes | NR | Yes | No | Good(12) |
| Stephen Murphy, 2015 (6) | Yes | Yes | CD | Yes | No | Yes | Yes | Yes | CD | Yes | CD | NR | CD | Yes | Fair (8) |
| James M. G. Curneen,2022(7) | Yes | Yes | Yes | Yes | Yes | Yes | Yes | Yes | Yes | Yes | Yes | NR | No | Yes | Good (12) |
| Paul Dillon,2019(8) | Yes | Yes | Yes | Yes | Yes | Yes | Yes | Yes | Yes | No | Yes | NR | Yes | Yes | Good(12) |
| Caroline A. Walsh, 2019(9) | Yes | Yes | Yes | No | Yes | Yes | Yes | Yes | Yes | CD | Yes | NR | Yes | Yes | Good (11) |
| Margaret Bermingham, 2011 (10) | Yes | Yes | Yes | Yes | Yes | Yes | Yes | Yes | Yes | Yes | Yes | NR | NA | Yes | Good (12) |
| Danielle Ní Chróinín, 2018 (11) | Yes | Yes | Yes | Yes | Yes | Yes | Yes | Yes | Yes | No | Yes | NA | NR | Yes | Good (11) |
| Catriona Murphy, 2015 (12) | Yes | Yes | Yes | Yes | Yes | Yes | No | Yes | yes | NA | Yes | NR | NA | Yes | Fair (10) |
| Kornelia Kotsevaa,2009(13) | Yes | Yes | CD | No | Yes | Yes | No | Yes | Yes | CD | Yes | NR | NA | No | Fair (7) |
| Ifeanyi Okechukwu,2007 (14) | Yes | Yes | Yes | Yes | Yes | CD | Yes | CD | Yes | No | Yes | CD | NA | Yes | Fair (9) |
| Kornelia Kotseva,2016(15) | Yes | Yes | Yes | No | No | Yes | No | Yes | Yes | NA | Yes | NR | Yes | No | Fair (8) |
| K. Kotseva,2007 (16) | Yes | Yes | Yes | Yes | Yes | Yes | No | Yes | Yes | NA | Yes | NR | NA | Yes | Fair (10) |
| Renata Cı´fkova´,2019 (17) | Yes | Yes | Yes | No | Yes | Yes | No | Yes | Yes | Yes | Yes | NA | NR | Yes | Fair (9) |
| Anselm K. Gitt, 2016(18) | Yes | Yes | Yes | No | Yes | Yes | No | Yes | Yes | NA | Yes | NR | NA | No | Fair (8) |
| Brendan Buckley,2009(19) | Yes | Yes | Yes | Yes | Yes | Yes | No | Yes | Yes | CD | Yes | CD | Yes | Yes | Good  (11) |
| Conor McCaughey, 2022 (20) | Yes | Yes | NR | Yes | Yes | Yes | Yes | Yes | Yes | CD | Yes | NR | NA | No | Fair (9) |
| A.A Syed, 2010(21) | Yes | Yes | Yes | Yes | No | Yes | CD | No | No | No | Yes | NR | NA | No | Fair (6) |
| Keating,2022(22) | Yes | Yes | No | Yes | Yes | Yes | Yes | Yes | CD | No | CD | NR | NA | No | Fair (7) |
| Kotseva, 2001(23) | Yes | Yes | Yes | No | No | Yes | No | Yes | Yes | NA | Yes | NR | NA | Yes | Fair (8) |

**Quality was rated for poor (0–4 out of 14 questions), for fair (5–10 out of 14 questions), or for good (11–14 out of 14 questions); NA: not applicable, NR: not reported (24,25).**

Reference:

1. Offiah G, O’Connor C, Kennedy C, Gallagher J, O’Connor P, McAdam B, et al. The DA VINCI study: is Ireland achieving ESC/EAS guideline-directed LDL-C goals? Ir J Med Sci [Internet]. 2022 Jul 1; Available from: https://www.scopus.com/inward/record.uri?eid=2-s2.0-85133257089&doi=10.1007%2fs11845-022-03050-6&partnerID=40&md5=66d49f0377acba1c047ce56ac51294cc

2. Dunne M, Mac Ananey O, Markham C, Maher V. Lipid targets in clinical practice: Successes, failures and lessons to be learned. Irish Journal of Medical Science. 2013;182(4):673–8.

3. Hayes P, Casey M, Glynn LG, Molloy GJ, Durand H, O’Brien E, et al. Measuring adherence to therapy in apparent treatment-resistant hypertension: a feasibility study in Irish primary care. Br J Gen Pract. 2019 Sep;69(686):e621–8.

4. Dillon P, Smith SM, Gallagher P, Cousins G. Impact of financial burden, resulting from prescription co-payments, on antihypertensive medication adherence in an older publically insured population. BMC Public Health. 2018 Nov 20;18(1):1282.

5. Kim S, Bennett K, Wallace E, Fahey T, Cahir C. Measuring medication adherence in older community-dwelling patients with multimorbidity. Eur J Clin Pharmacol. 2018 Mar;74(3):357–64.

6. Murphy SJX, Coughlan CA, Tobin O, Kinsella J, Lonergan R, Gutkin M, et al. Continuation and adherence rates on initially-prescribed intensive secondary prevention therapy after Rapid Access Stroke Prevention (RASP) service assessment. J Neurol Sci. 2016 Feb 15;361:13–8.

7. Curneen JMG, Rabbitt L, Browne D, O’Donoghue DF, Alansari Y, Harhen B, et al. Major disparities in patient-reported adherence compared to objective assessment of adherence using mass spectrometry: A prospective study in a tertiary-referral hypertension clinic. Br J Clin Pharmacol. 2022 Feb 23;

8. Dillon P, Smith SM, Gallagher P, Cousins G. The association between pharmacy refill-adherence metrics and healthcare utilisation: a prospective cohort study of older hypertensive adults. Int J Pharm Pract. 2019 Oct;27(5):459–67.

9. Walsh CA, Cahir C, Bennett KE. Association between adherence to antihypertensive medications and health outcomes in middle and older aged community dwelling adults; results from the Irish longitudinal study on ageing. Eur J Clin Pharmacol. 2019 Sep;75(9):1283–92.

10. Bermingham M, Hayden J, Dawkins I, Miwa S, Gibson D, McDonald K, et al. Prospective analysis of LDL-C goal achievement and self-reported medication adherence among statin users in primary care. Clin Ther. 2011 Sep;33(9):1180–9.

11. Ní Chróinín D, Ní Chróinín C, Akijian L, Callaly EL, Hannon N, Kelly L, et al. Suboptimal lipid management before and after ischaemic stroke and TIA-the North Dublin Population Stroke Study. Ir J Med Sci. 2018 Aug;187(3):739–46.

12. Murphy C, Bennett K, Fahey T, Shelley E, Graham I, Kenny RA. Statin use in adults at high risk of cardiovascular disease mortality: Cross-sectional analysis of baseline data from The Irish Longitudinal Study on Ageing (TILDA). BMJ Open [Internet]. 2015;5(7). Available from: https://www.scopus.com/inward/record.uri?eid=2-s2.0-84937197924&doi=10.1136%2fbmjopen-2015-008017&partnerID=40&md5=d8f841a657414cd52d4a417bdbc9e3ad

13. Kotseva K, Wood D, De Backer G, De Bacquer D, Pyörälä K, Keil U. EUROASPIRE III: A survey on the lifestyle, risk factors and use of cardioprotective drug therapies in coronary patients from 22 European countries. European Journal of Cardiovascular Prevention and Rehabilitation. 2009;16(2):121–37.

14. Okechukwu I, Mahmud A, Bennett K, Feely J. Choice of first antihypertensive--are existing guidelines ignored? Br J Clin Pharmacol. 2007 Dec;64(6):722–5.

15. Kotseva K, Wood D, De Bacquer D, De Backer G, Rydén L, Jennings C, et al. EUROASPIRE IV: A European Society of Cardiology survey on the lifestyle, risk factor and therapeutic management of coronary patients from 24 European countries. Eur J Prev Cardiol. 2016 Apr;23(6):636–48.

16. Kotseva K, Stagmo M, De Bacquer D, De Backer G, Wood D. Treatment potential for cholesterol management in patients with coronary heart disease in 15 European countries: Findings from the EUROASPIRE II survey. Atherosclerosis. 2008;197(2):710–7.

17. Cífková R, Lánská V, Bruthans J, Mayer O, Kotseva K, Wood D, et al. Blood pressure distribution and control in coronary patients from 24 European countries in the European Society of Cardiology EURoObservational Research Programme European survey of cardiovascular disease prevention and diabetes. EUROASPIRE IV Registry. Journal of Hypertension. 2019;37(10):2015–23.

18. Gitt AK, Lautsch D, Ferrieres J, Kastelein J, Drexel H, Horack M, et al. Contemporary data on low-density lipoprotein cholesterol target value attainment and distance to target in a cohort of 57,885 statin-treated patients by country and region across the world. Data in Brief. 2016 Dec 1;9:616–20.

19. Buckley B, Shanahan E, Colwell N, Turgonyi E, Bramlage P, Perry IJ. Blood pressure control in hypertensive patients in Irish primary care practices. J Clin Hypertens (Greenwich). 2009 Aug;11(8):432–40.

20. McCaughey C, Ranganathan D, Kerins M, Murphy G, Murphy R. Dyslipidaemia management in the cardiac rehabilitation clinic of a tertiary referral centre: analysis of the impact of new ESC guidance on LDL-C target achievement. Ir J Med Sci. 2022 Dec;191(6):2569–77.

21. Syed IAA, Riaz A, Ryan A, Reilly MO. Secondary prevention for coronary artery disease: are we following the guidelines? Ir J Med Sci. 2010 Dec;179(4):535–7.

22. Keating T, AlAdalieh M, Chughtai Z, Javadpour SH. Adherence to secondary prevention recommendations after coronary artery bypass graft surgery. Ir J Med Sci. 2022 Aug 25;1–6.

23. Lifestyle and risk factor management and use of drug therapies in coronary patients from 15 countries; principal results from EUROASPIRE II Euro Heart Survey Programme. Eur Heart J. 2001 Apr;22(7):554–72.

24. Bagias C, Sukumar N, Weldeselassie Y, Oyebode O, Saravanan P. Cord Blood Adipocytokines and Body Composition in Early Childhood: A Systematic Review and Meta-Analysis. International Journal of Environmental Research and Public Health. 2021;18(4).

25. the National Heart, Lung, and Blood Institute Quality Assessment Tool for Observational Cohort and Cross-sectional Studies. (Accessed May 2023) [Internet]. Available from: https://www.nhlbi.nih.gov/health-topics/study-quality-assessment-tools
